# Clinico-histopathological and phylogenetic analysis of protozoan epibiont *Epistylis wuhanensis* associated with crustacean parasite *Lernaea cyprinacea* from ornamental fish in Iran

**DOI:** 10.1038/s41598-023-41368-y

**Published:** 2023-08-28

**Authors:** Hooman Rahmati-Holasoo, Amin Marandi, Sara Shokrpoor, Taranom Goodarzi, Zahra Ziafati Kafi, Iradj Ashrafi Tamai, Hosseinali Ebrahimzadeh Mousavi

**Affiliations:** 1 Department of Aquatic Animal Health, Faculty of Veterinary Medicine, University of Tehran, Tehran, Iran; 2 Department of Pathology, Faculty of Veterinary Medicine, University of Tehran, Tehran, Iran; 3 Faculty of Veterinary Medicine, University of Tehran, Tehran, Iran; 4 Department of Microbiology and Immunology, Faculty of Veterinary Medicine, University of Tehran, Tehran, Iran

**Keywords:** Parasitology, Pathogens

## Abstract

Parasitic infestations are one of the most concerning problems limiting ornamental fish farming. In addition to the direct economic losses due to the major mortalities, parasites may significantly negatively impact the body shape, coloration, growth rate, and health condition of the fish. The results of the study highlight the importance of comprehensive parasitological analysis in the diagnosis and treatment of parasitic infections in ornamental fish farms. The presence of multiple parasites in the affected fish emphasizes the need for effective biosecurity measures, such as extending the quarantine period for newly imported fish, closely monitoring fish populations, and implementing isolation units to prevent the spread of infections. By implementing these preventative measures, ornamental fish farmers can reduce the risk of parasitic infections and ensure the health and well-being of their fish populations. This, in turn, can lead to increased profitability and sustainability for their business. Overall, the current study aimed to conduct a clinical, histopathological, and phylogenetic analysis of the epibiont ciliated protozoan *Epistylis wuhanensis* and the copepod crustacean *Lernaea cyprinacea* in a freshwater ornamental fish farm in Iran. Furthermore, it provides valuable insights into the prevalence and impact of parasitic infections in ornamental fish farms and underscores the need for continued research and the development of effective preventative measures to address this issue. A total of 60 symptomatic freshwater ornamental fish, including 30 guppy (*Poecilia reticulata*) and 30 sailfin molly (*Poecilia latipinna*), were packed in polyethylene bags filled with oxygenated pond water and transported to the Ornamental Fish Clinic, Faculty of Veterinary Medicine, University of Tehran, for parasitological analysis. Following the clinical examination, histopathological analysis was performed on 10% NBF (neutral buffered formalin)-fixed samples from affected tissues, including the skin, skeletal muscle, and liver, to identify any pathological changes associated with the parasitic infections. Furthermore, the DNA was extracted from the 99% ethanol-fixed samples using a commercial DNA extraction tissue kit (SinaPure DNA, Iran), and PCR was performed using Peri18S-F1 (5′-ACC TGG TTG ATC CTG CCA GT-3′) and Peri18S-R1 (5′-TGC AGG TTC ACC TAC GGA AA-3′) (first reaction), and Peri18S-F2 (5′-CCG CGG TAA TTC CAG CTC-3′) and Peri18S-R2 (5′-GAT CCC CTA ACT TTC GTT CTT GA-3′) (second round) primers for the identified parasites. Finally, the PCR products were sequenced using Sanger dideoxy sequencing methods, and the resulting sequences were compared to sequences in the BLAST search program to provide a comprehensive picture of the current parasite-based disorder. The crustacean *L. cyprinacea* and the epibiont sessilid *E. wuhanensis* were identified in the examined ornamental guppy (6/30) and sailfin molly (6/30), with an overall parasitic prevalence of 20.00% (12/60). Ciliates were found in all tissue lesions but not in fish without lesions. A great number of the ciliated protozoan *E. wuhanensis* were found attached to the integumentary area of *L. cyprinacea*. Microscopically, oval to round granulomatous lesions were observed in cutaneous and skeletal muscles. Lymphoplasmacytic dermatitis and myositis were also observed. The crustacean *L. cyprinacea* serves as a mechanical vector for *E. wuhanensis* infection and spreads the disease in ornamental fish farming operations. For the first time in Iran, we successfully presented diagnostic morphological and molecular data on sessilids isolated from *L. cyprinacea*. Based on the findings of the current study, such parasitic infections may cause significant economic losses following invasion of the integument area of fish, eventually leading to death, if treatment is neglected or inadequate. Furthermore, the findings of the analysis were used to develop effective diagnostic approaches for the affected fish, as well as recommendations for improved health conditions to prevent future outbreaks of parasitic infections. However, further research is needed to determine the precise mechanisms of crustacean attachment and host-crustacean-peritrich protozoan interactions. Furthermore, the direct and indirect effects of various environmental factors on the emergence and spread of the current disease should be considered.

## Introduction

Ornamental fish farming is one of the industries that has grown significantly in recent years and benefits from a high annual turnover^1–3^. According to recent statistics on the top exporter continents of ornamental fish, Asia ranked first (57% of total world exports), as well as Singapore, Japan, the Czech Republic, Thailand, Malaysia, Indonesia, Israel, Brazil, Sri Lanka, and Columbia, which were announced as the top 10 exporters of ornamental fish. On the other hand, the United States, United Kingdom, Germany, Singapore, Japan, China, France, the Netherlands, Italy, and Malaysia were announced as the top 10 importers of ornamental fish^4^. Although a large portion of imported ornamental fish was previously allocated to East Asian countries^5^, the trade of ornamental fish has significantly improved in recent decades and has evolved into an economically profitable industry among Iranian enthusiasts. Iran has long been a major importer of ornamental fish, and the majority of its imports come from Japan, Singapore, Thailand, and Indonesia. However, in the last 20 years, the cultivation and breeding of ornamental fish have increased dramatically^6–9^.

Parasitic infestations are one of the most concerning issues limiting ornamental fish farming and, as a result, affecting the global aquaculture industry^6,7,10,11^. Essentially, transferring freshwater ornamental fish from the wild to size-limited environments may increase the risk of parasitic epidemics in ornamental fish farms. Furthermore, failure to adhere to hygienic protocols during the temporary maintenance period of imported fish in wholesalers’ and importers units may be regarded as a booster for the occurrence of parasitic diseases^12,13^. Some parasites only infiltrate the outer surfaces of the various organs. Other parasites, on the other hand, can penetrate the parenchyma of the host’s various tissues^14^. Parasites may have a significant negative impact on the body shape, body weight, coloration, growth rate^15,16^, health condition^17^, and reproductive function of the fish, in addition to direct economic losses due to major mortalities^16,18^.

Copepod crustaceans are one of the most significant parasitic pathogens^17,19^ found in ornamental fish farms and natural habitats^17,20,21^. *Lernaea cyprinacea* Linnaeus, 1758, also known as the anchor worm, is a highly modified and widely distributed copepod crustacean in the Lernaeidae family, which includes more than 14 genera and 110 species^22^. As a relatively immobile parasite, *L. cyprinacea* is capable of attaching to the gills as well as the skin^23^, causing tissue lesions such as epithelial hyperplasia, telangiectasis, hemorrhage^24^, and gill epithelium necrosis and disruption, which can lead to death in both marine and freshwater ornamental fish^22,23^. Because of the increasing prevalence of this parasite in various species of ornamental fish all over the world, the economic importance of the crustacean *L. cyprinacea* has been increasingly considered^25^. *Lernaea cyprinacea*^17,26–29^ and other parasitic crustaceans^30–32^ may also provide substrate for epibionts such as *Epistylis*. The ability to be passively transported with a clear increase in feeding, improvement of food capturing efficiency^33^ and feeding rate^34^, and a decrease in predation^35,36^ are the main benefits for epibionts.

Despite the occasional reports of parasitic infections caused by peritrich ciliate attachment to the anchor worm, in a wide range of freshwater fish species in different geographical regions of the world (Table 1), the present study aimed to fill the knowledge gap regarding parasitic infections caused by the attachment of peritrich ciliates to *L. cyprinacea* in ornamental fish farms in the Middle East, specifically in Iran. The study used a combination of clinical, histopathological, and phylogenetic analyses to identify and characterize the parasites in question. By doing so, the study aimed to provide a better understanding of the prevalence, pathology, and genetic relatedness of these parasites in ornamental fish farms in the region.

**Table 1:** Records of peritrich ciliates attached to *L. cyprinacea* in different species of fish.

Host fish	Peritrich ciliate	Attachment location in fish	Geographical region	Reference
*Pelteobagrus fulvidraco*	*Epistylis wuhanensis*	Fins	China	Wang et al.^27^
*Rhinogobius similis*	*Epistylis wuhanensis*	Body surface	Japan	Nagasawa and Nitta^37^, Nitta^29^
	Vorticellidae gen. sp.	–	Japan	Fukushima et al.^38^
*Rhinogobius brunneus*	Vorticellidae gen. sp.	–	Japan	Nagasawa et al.^39^
*Rhinogobius nagoyae*	Vorticellidae gen. sp.	–	Japan	Fukushima et al.^38^
*Tridentiger brevispinis*	Vorticellidae gen. sp.	–	Japan	Fukushima et al.^38^
*Xiphophorus helleri*	Vorticellidae gen. sp.	–	Japan	Fukushima et al.^38^
*Megaleporinus obtusidens*	*Epistylis* sp.	Gills	Brazil	Pala et al.^17^
*Lepomis macrochirus*	Peritrich ciliates	–	Japan	Grygier^40^
*Squalidus gracilis gracilis*	Peritrich ciliates	–	Japan	Nagasawa et al.^41^
*Oryzias latipes*	Peritrich ciliates	–	Japan	Nagasawa et al.^42^
	Peritrich ciliates	–	Japan	Nagasawa et al.^43^
	Peritrich ciliates	–	Japan	Nagasawa et al.^44^
	Peritrich-like ciliates	–	Japan	Beatte et al.^45^
	Peritrich-like ciliates	–	Japan	Nagasawa et al.^46^
*Poecilia reticulata*	*Epistylis wuhanensis*	Body surface & muscle	Iran	Present study
*Poecilia latipinna*	*Epistylis wuhanensis*	Body surface & muscle	Iran	Present study

## Methods

### Fish and sampling

**Figure 1:**
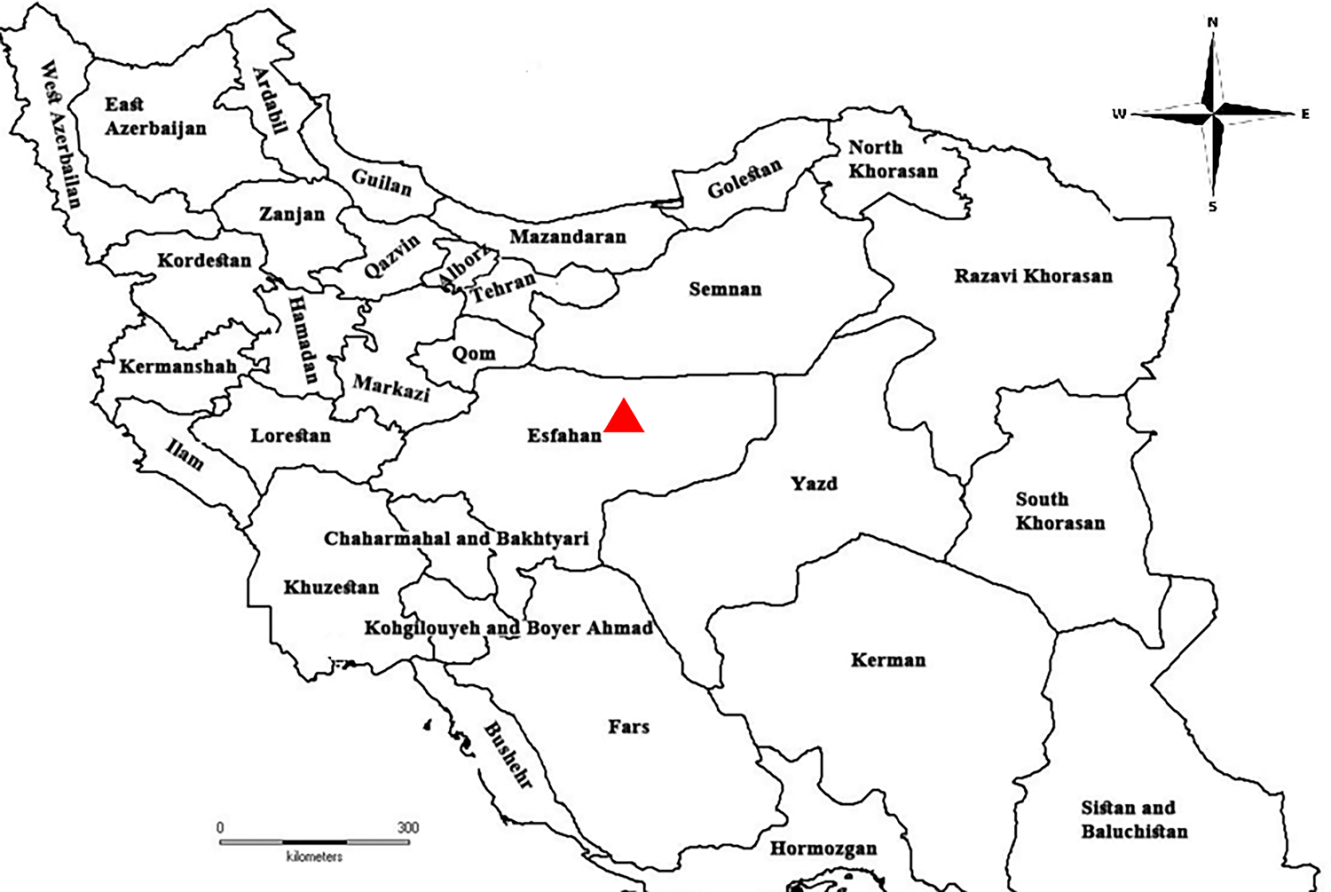
Sampling location of freshwater ornamental guppy (*Poecilia reticulata*) and sailfin molly (*Poecilia latipinna*) farm (black filled triangle) in Esfahan province in Iran (33.3250° N, 53.3906° E) (The map has been modified from Rahmati-Holasoo, H., Marandi, A., Ebrahimzadeh Mousavi et al*.* Parasitic fauna of farmed freshwater ornamental fish in the northwest of Iran. *Aquacult Int* **30**, 633–652 (2022). https://doi.org/10.1007/s10499-021-00832-0).

During the period from October 2020 to September 2021, listlessness, anorexia, weakness, and the flashing of guppy (*Poecilia reticulata*) and sailfin molly (*Poecilia latipinna*) were accompanied by mass mortalities (31% and 42%, respectively), leading to significant economic losses in an ornamental fish farm located in the Esfahan province of Iran (33.3250° N, 53.3906° E) (Fig. 1). A total of 60 symptomatic freshwater ornamental fish, including 30 guppy and 30 sailfin molly (a length of 2–2.5 cm), were packed in polyethylene bags filled with oxygenated pond water and transported to the Ornamental Fish Clinic, Faculty of Veterinary Medicine, University of Tehran (Tehran, Iran), for a preliminary and subsequent complementary parasitological analysis.

### Parasitological analysis

**Figure 2:**
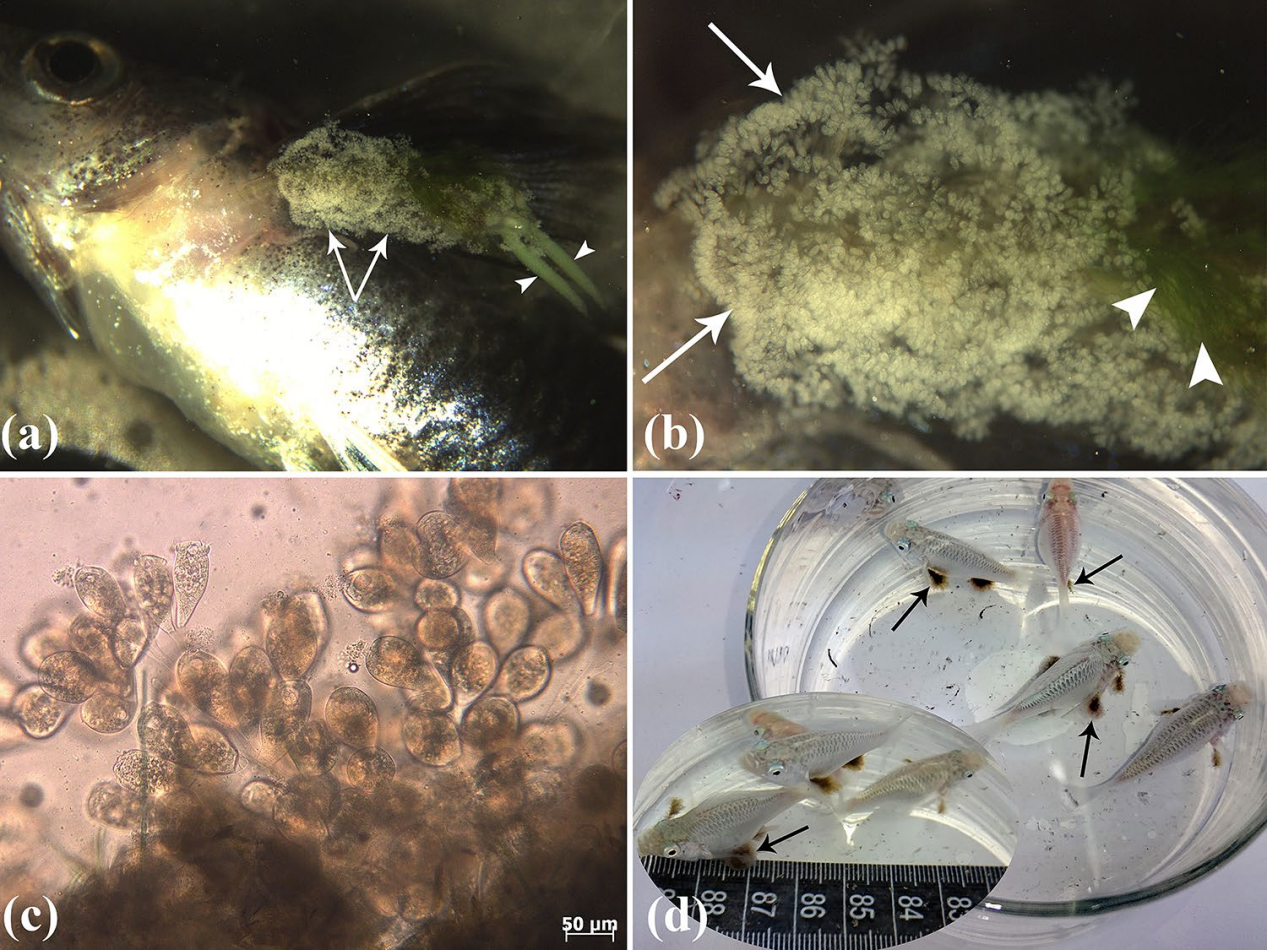
(**a**) A stereomicroscopic study of *Poecilia reticulata* that uses a stereoscope to do a more detailed inspection showing intense adhesion of *E. wuhanensis* (arrows) in the cephalothorax and trunk of the crustacean *L. cyprinacea*. Egg sacs of *L. cyprinacea* (arrowheads) are also visible (10X). (**b**) High-power view of the ciliated protozoan *E. wuhanensis* (arrows) adhered to *L. cyprinacea* on the body surface of *P. reticulata*. Note the algae (arrowheads) adhered to *L. cyprinacea* and *E. wuhanensis* (40X). (**c**) Wet mount preparation from *P. reticulata* showed infestation with a large number of the epibiont ciliated protozoan *E. wuhanensis*. (**d**) Macroscopic examination of *Poecilia latipinna* showed infestation with* E. wuhanensis* adhered to *L. cyprinacea* (arrows).

**Figure 3:**
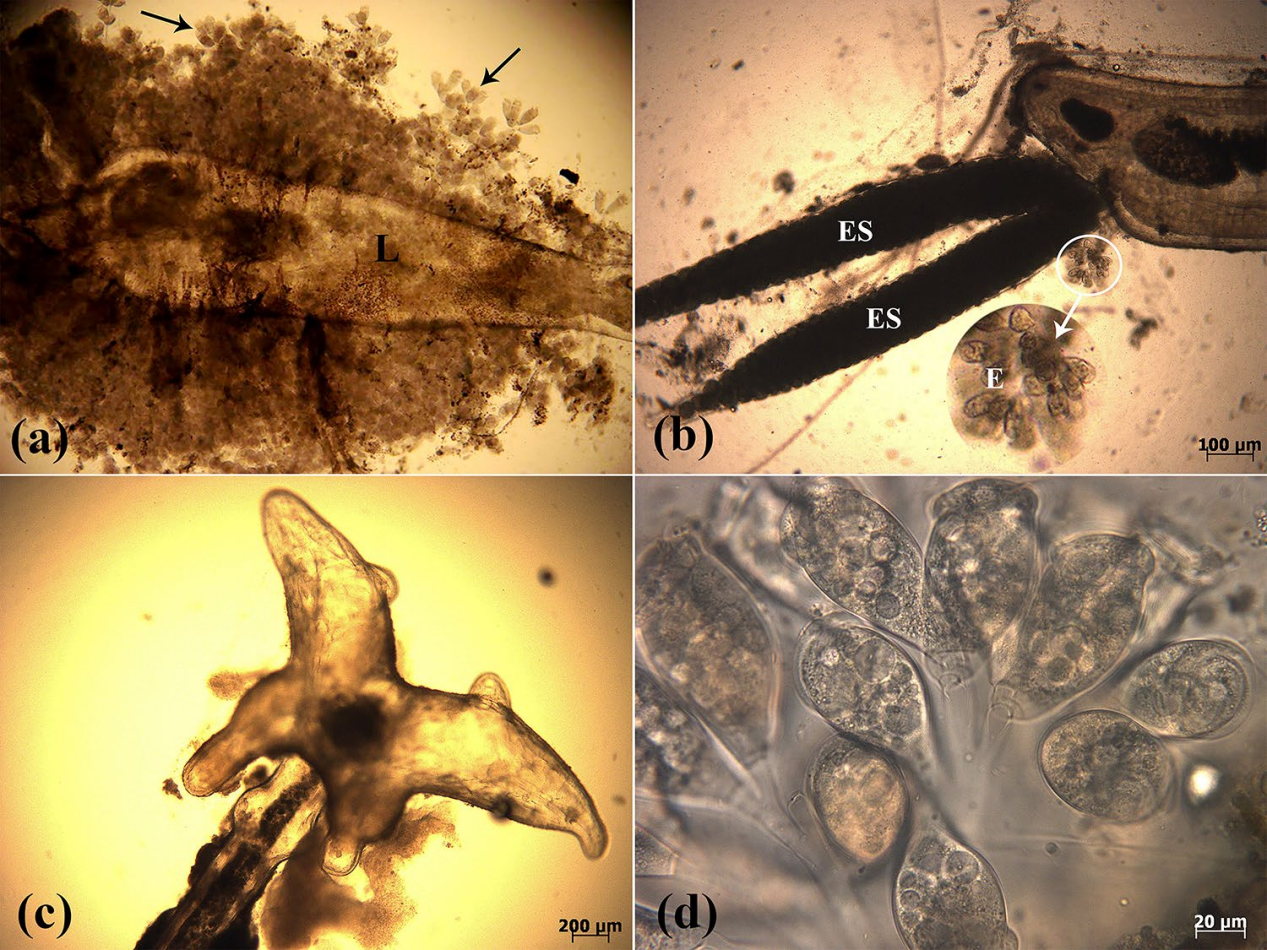
(**a**) Wet mount preparation from *P. latipinna* showed infestation with a large number of the ciliated protozoan *E. wuhanensis* (arrows), which were found attached severely to the integumentary area of the crustacean *L. cyprinacea* (L). (**b**) Wet mount preparation of *P. latipinna* revealed the ciliated protozoan *E. wuhanensis* (E) attached to the egg sacs (ES) of *L. cyprinacea*. (**c**) Wet mount preparation of *P. latipinna* revealed the antenna and anchors of *L. cyprinacea*. (**d**) Wet mount preparation from *P. reticulata* showed infestation with the ciliated protozoan *E. wuhanensis*. Note the mature zooid of *E. wuhanensis* showing oral ciliature.

**Figure 4:**
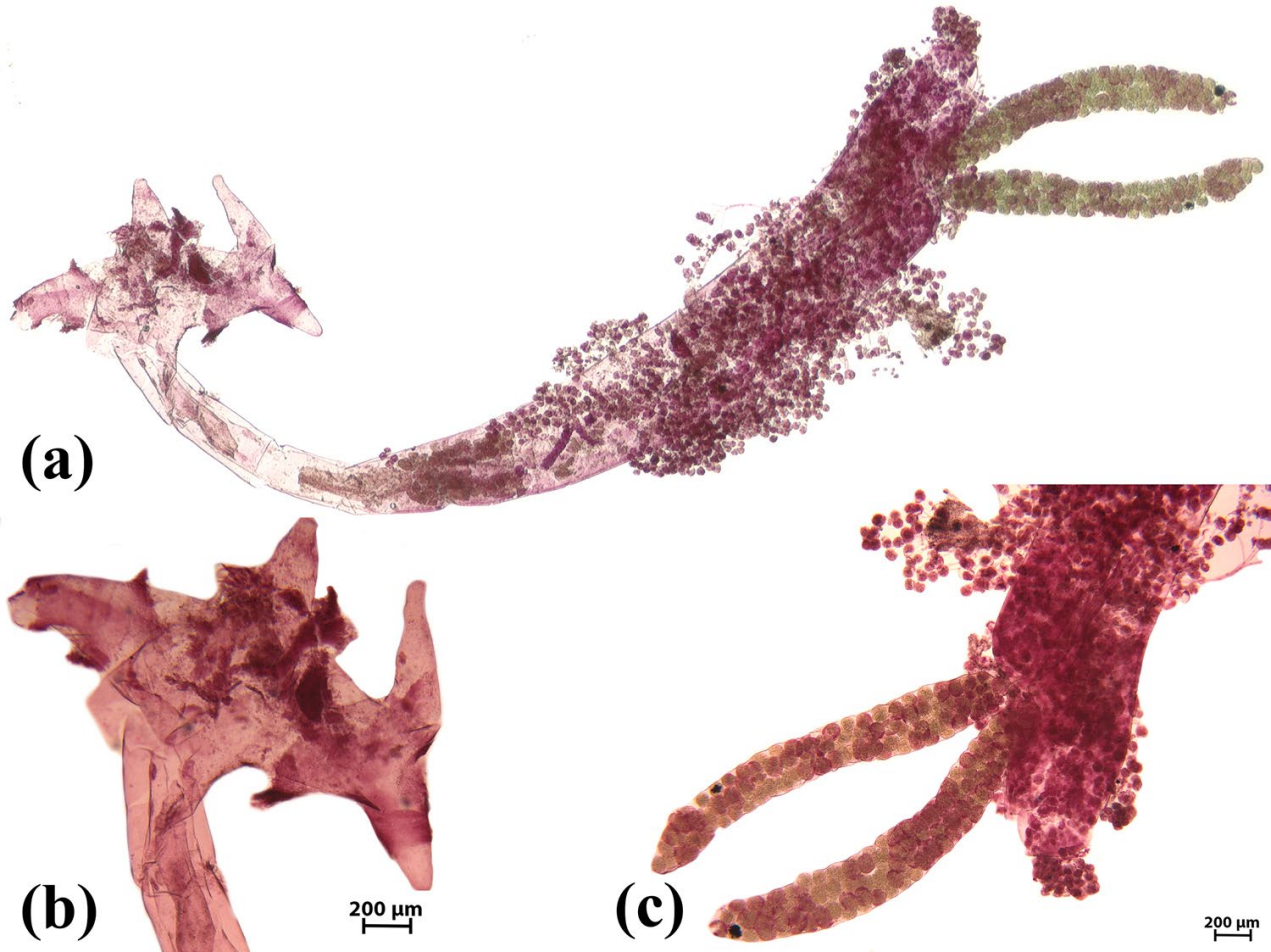
Scanning images of carmen-stained sections of the crustacean *L. cyprinacea* isolated from the body surface of *P. laptinana*. Note the great number of *E. wuhanensis* attached to *L. cyprinacea*. (**a**) Lateral view. (**b**) Lateral view of the cephalothorax and anchors. (**c**) Lateral view of egg sacs.

A thorough macroscopic examination of the body surface and fins performed at the Ornamental Fish Clinic to verify any parasites, lesions, or alterations revealed the clear attachment of hookworms to the external surfaces of the fish (Fig. 2a,d). Following the owner’s consent, fish were anaesthetized in 100 ppm PI222 (the major active ingredients of which are eugenol, carvacrol, and eugenol acetate) (Pars Imen Daru, Iran), and wet mounts of scrapings (of the body surface and dorsal, pectoral, ventral, and caudal fins) were prepared and used for microscopic observation of parasites under a light microscope (Nikon E600, Japan) (Figs. 2c and 3a–d) and a trinocular stereomicroscope (Olympus SZ60, Japan) (Fig. 2a,b). A microscopic examination of fish revealed some peritrich ciliated protozoans that were firmly attached to copepod crustaceans. The use of fresh fish samples may aid in the visualization of peritrich parasite motivation^37,38^. Copepod crustaceans were clarified with lactophenol and identified according to keys described by Damaree^39^, and Lester and Hayward^23^. In addition, the Plustek OpticLab H850 slide scanner was used to scan carmen-stained sections of the crustacean *L. cyprinacea* and attached *E. wuhanensis* (Fig. 4a–c).

### Histopathological analysis

For histological examinations, integumentary lesions of fish were dissected and fixed in 10% neutral buffered formalin, dehydrated in an ethanol series, and embedded in paraffin with a paraffin tissue processor and paraffin dispenser. Several sections were cut at 4 µm, and stained with haematoxylin–eosin (H&E). Sections were examined by light microscopy (Nikon E600, Japan), and representative images were taken using an IDS UI-2250 microscope camera (IDS imaging).

### PCR detection

Total genomic DNA was extracted from 99% ethanol-fixed specimens using a DNA extraction tissue kit (SinaPure DNA, Iran) according to the manufacturer's instructions. The amplification reaction was performed using a nested PCR assay for conserved regions that maximizes coverage of the included 18S region and breaks it into two overlapping segments. PCR was performed in a total volume of 25 µl, containing 12.5 µl of Master Mix (Amplicon, Denmark), 1 µl of each 10 pM primer, 2 µl of extracted DNA, and 8.5 µl of distilled water.

Peri18S-F1 (5′-ACC TGG TTG ATC CTG CCA GT-3′) and Peri18S-R1 (5′-TGC AGG TTC ACC TAC GGA AA-3′) were used in the first reaction, and Peri18S-F2 (5′-CCG CGG TAA TTC CAG CTC-3′) and Peri18S-R2 (5′-GAT CCC CTA ACT TTC GTT CTT GA-3′) were the primers used in the second round^40^. Part of the ribosomal region incorporating the internal transcribed spacers 1 and 2 and the 5.8S rDNA (ITS1-5.8S-ITS2) gene was amplified using the primer pairs ITS-F (5′-GTA GGT GAA CCT GCG GAA GGA TCA TTA-3′) and ITS-R (5′-TAC TGA TAT GCT TAA GTT CAG CGG-3′)^29,41^.

The cycling conditions included an initial denaturation at 94 °C for 5 min, followed by 35 cycles at 94 °C for 30 s, 54 °C for 30 s, 72 °C for 60 s, and 72 °C for 5 min as the final extension.

The amplification products (5 µl) were resolved by electrophoresis on a 1.5% agarose gel in 1X TBE buffer for 1 h at 100 V. Afterwards, the agarose gel was stained with 1 µg/ml ethidium bromide (CinnaGen, Iran) to visualize PCR Products under UV light. The gel was screened using a UV-transilluminator (BIORAD, UK) to visualize the DNA fragments. The presence of the DNA was confirmed by comparing the size of the PCR products with a DNA ladder of known sizes run alongside the PCR products on the gel.

### Phylogeny and sequence analysis

The phylogenetic analysis was performed to determine the evolutionary relationships between the strains of *L. cyprinacea* and *Epistylis wuhanensis* isolated from guppy (*Poecilia reticulata*) and sailfin molly (*Poecilia latipinna*). The strains were sequenced by the Macrogen Company in South Korea, and Sanger dideoxy sequencing methods were used to obtain these sequences. Analyzed individually using BioEdit version 7 was used for gene annotation and sequence trimming^42^. High-quality sequences (determined by the size of the fluorescent signal and the clarity of the peak) obtained from the study, were analyzed using the BLAST search program (https://blast.ncbi.nlm.nih.gov/Blast.cgi), and submitted to BankIt. Bootstrap values were calculated in MEGA7 based on 1000 replicates to assess the statistical support for each node. The accession numbers are OP175983 for *L. cyprinacea* isolate RS13 and OP175994 for *E. wuhanensis* isolate RS14. Each of the gene sequences of Sessilids was retrieved from GenBank, and a FASTA dataset was generated. Multiple sequence alignments were generated by ClustalW and then used to generate distance matrices using the General Time Reversible (GTR) model implemented in MEGA software version 7^43,44^. Finally, the Maximum Likelihood (ML) trees were plotted by MEGA7 utilizing a 1,000-fold bootstrap approach, as prescribed by Kumar et al.^43^ and Nei and Kumar^44^.

### Ethics approval and consent to participate

In the current study, clinical records were provided following owner consent, and the data were securely stored. In addition, ethical approval for this study was granted by the University of Tehran Veterinary Ethical Review Committee. All methods were performed in accordance with the guidelines and regulations of the University of Tehran Veterinary Ethical Review Committee. Also, the study is reported in accordance with ARRIVE guidelines. Written informed consent was obtained from the owner for the participation of the animal in the study.

## Results

### Clinical findings and laboratory examinations

The crustacean *L. cyprinacea* and the epibiont sessilid ciliate *E. wuhanensis* were identified in the examined ornamental guppy (6/30) and sailfin molly (6/30), with an overall parasitic prevalence of 20.00% (12/60) (Fig. 2a,b,d). Ciliates were found in all tissue lesions but not in fish without lesions. These sessile ciliates were identified and assigned to the subclass Peritrichia based on morphological characteristics such as the absence of feeding tentacles and the presence of a cytopharynx. They were also classified as Epistylididae due to the presence of a contractile body, a non-contractile stalk, the absence of aboral arms, and the absence of a stalk on the peristomal disc (Fig. 3d). They were assigned to *Epistylis* due to their colonial nature and three-turning oral cilia (Fig. 3d). A great number of the ciliated protozoan *E. wuhanensis* were found attached to the integumentary area of *L. cyprinacea* (Figs. 2c and 3a,b).

### Histopathological findings

**Figure 5:**
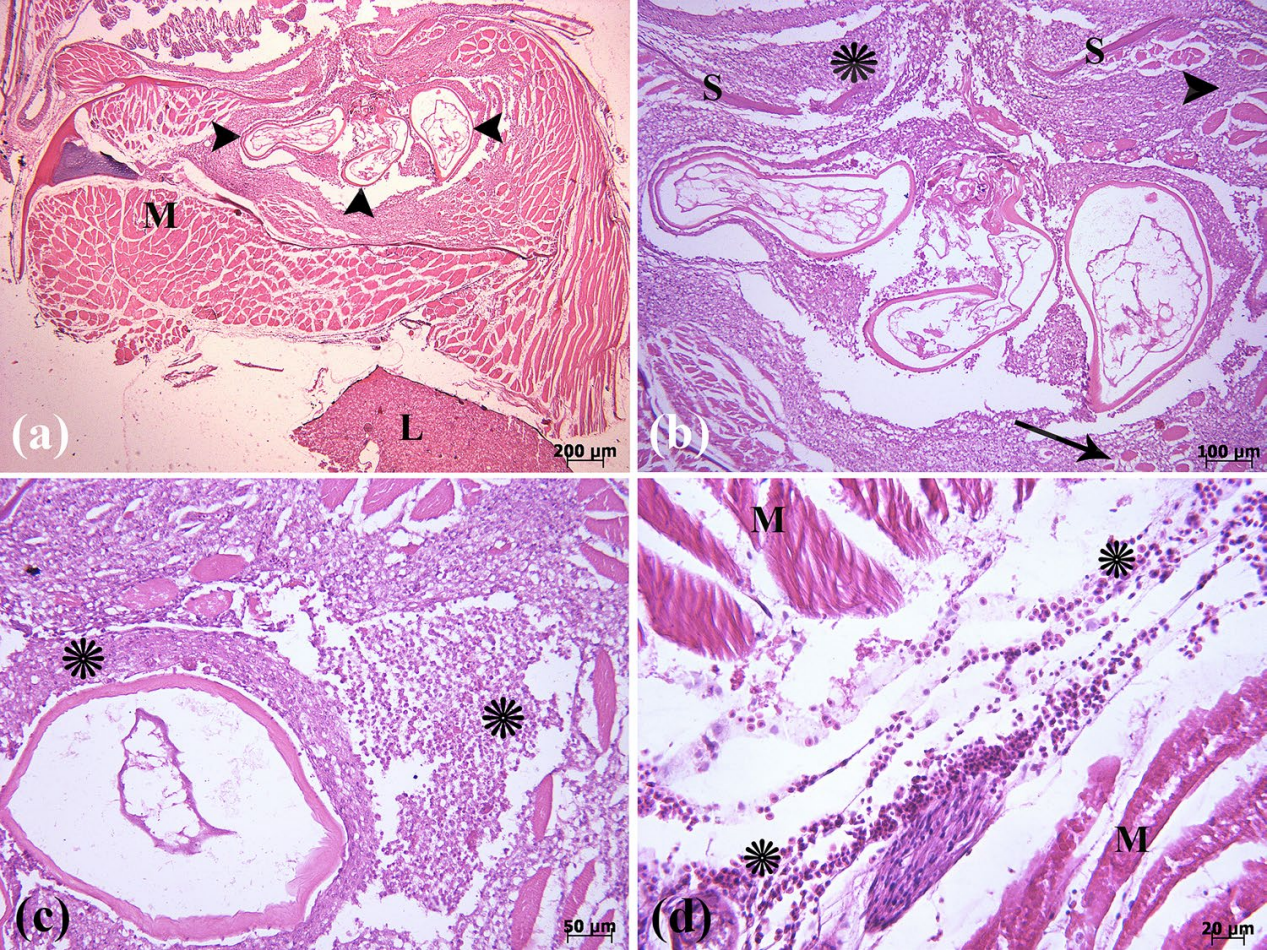
Histopathological findings of *Poecilia reticulata* and *Poecilia latipinna* due to infection by the crustacean parasite *L. cyprinacea* in skin and muscle tissue. (**a**) Triple cross sections of a parasite in granulomatous inflammation (arrowheads) in the dermal layer and skeletal muscle tissue, muscle (M) and liver (L). (**b**) Higher magnification of parasitic granuloma, lymphoplasmacytic dermatitis (*) and myositis (arrowhead), muscle fiber atrophy (arrow), and scale (S). (**c**) Inflammatory cells (particularly lymphocytes, plasma cells, macrophages, and eosinophils) (*) congregate around the parasite. (**d**) Hemorrhage (*) is observed in the skeletal muscle (M). (H&E).

**Figure 6:**
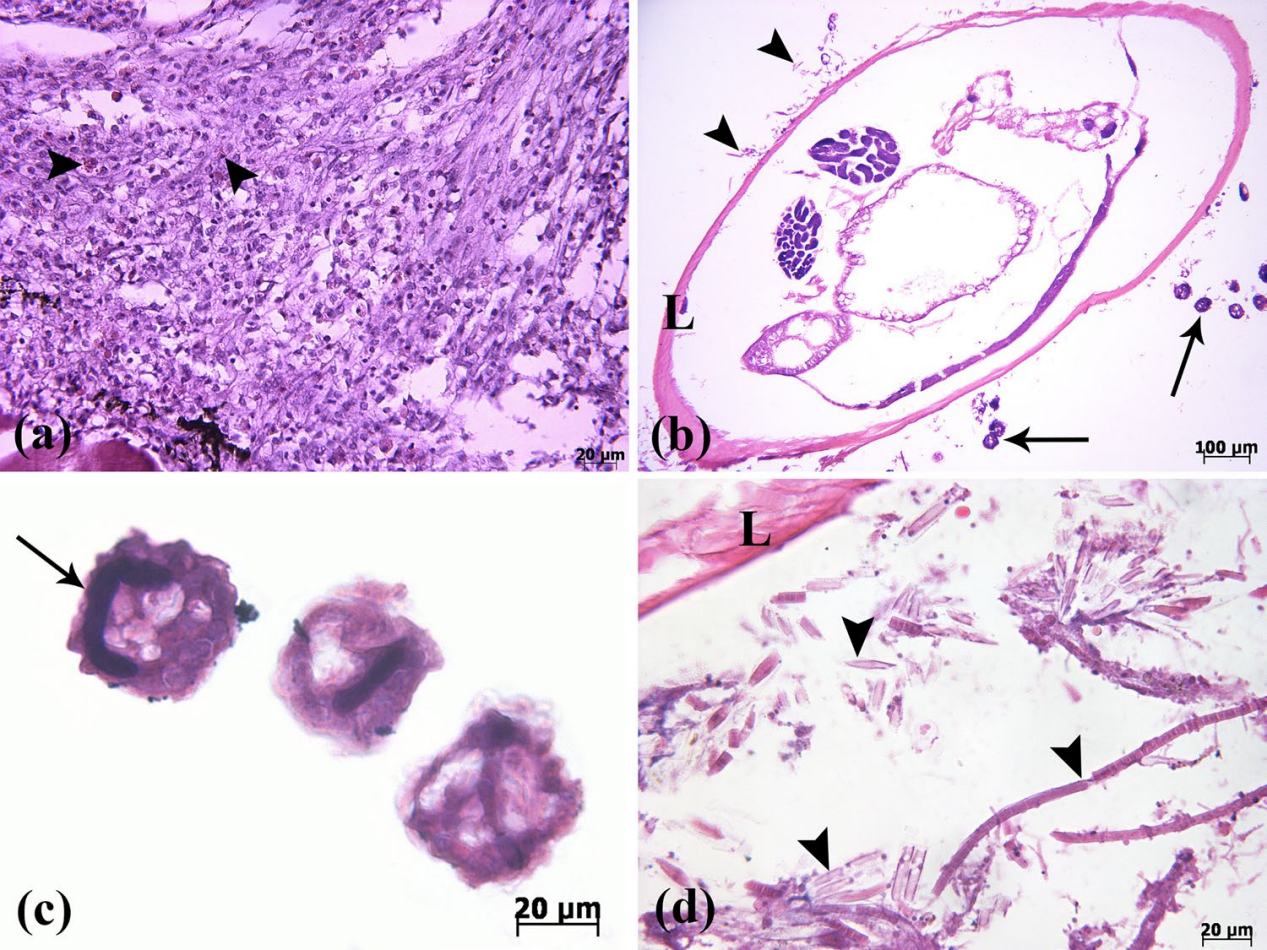
(**a**) The accumulation of eosinophils (arrowheads) around the parasite in a parasitic eosinophilic granuloma. (**b**) The transverse-section of *L. cyprinacea* (L), *E. wuhanensis* (arrows), and algae (arrowheads). (**c**) In detail, the transverse-section of *E. wuhanensis*. Horseshoe-shaped macronucleus of the parasite is shown (arrow). (**d**) Different morphological types of algae (arrowheads) around the crustacean parasite *L. cyprinacea* (L) (H&E).

Tissue lesions of variable severity were present in all parasitized fish. Microscopically, oval to round granulomatous lesions were observed in cutaneous and skeletal muscles. These lesions caused pressure atrophy of the adjacent skeletal muscle fibers. Lymphoplasmacytic dermatitis and myositis were also observed (Fig. 5a,b). In granulomatous structures, cross sections of parasite and necrotic cell debris were seen in the central part. The parasite was surrounded by a dense zone of inflammatory cells. There were macrophages, lymphocytes, plasma cells, and eosinophils in the inner area (Figs. 5c and 6a). The outermost area surrounding the granuloma consisted of fibroblasts (Fig. 5c). Hemorrhage was also observed in skeletal muscle around the granulomas (Fig. 5d). Finally, a chronic and parasitic eosinophilic granulomatous inflammation was diagnosed. Adhesion of *E. wuhanensis* and algae in the cephalothorax and trunk of the crustacean *L. cyprinacea* was observed in histological sections (Fig. 6b–d).

### Molecular findings

**Figure 7:**
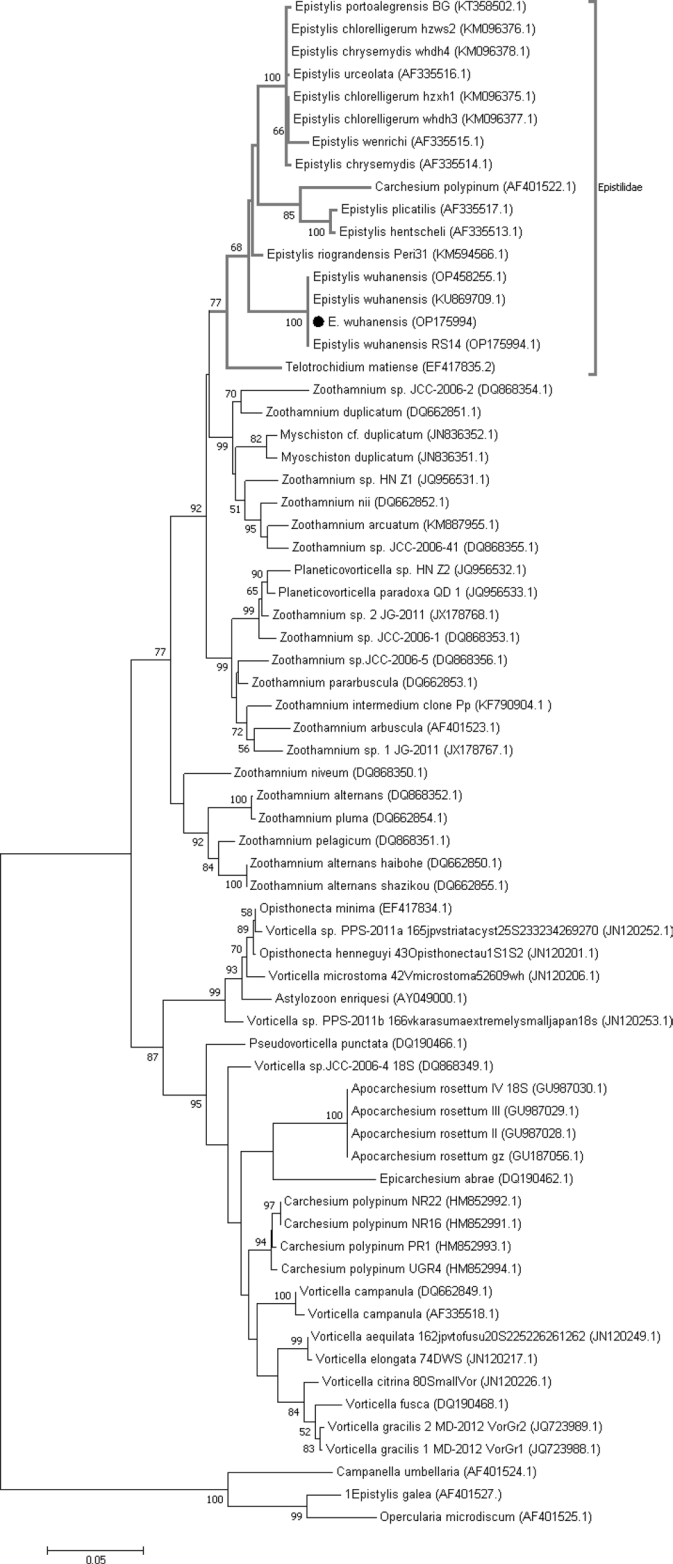
Molecular phylogenetic analysis based on the nucleotide sequences of the partial 18 s gene using the maximum likelihood method based on the general time-reversible model^44^. Evolutionary analyses were performed in MEGA7^43^. The bootstrap consensus tree derived from 1000 replicates represents the evolutionary history of the taxa analyzed. The percentage of trees in which the associated taxa were clustered together is indicated next to the branches. The tree is drawn to scale, with the length of branches measured in the number of substitutions per site.

**Figure 8:**
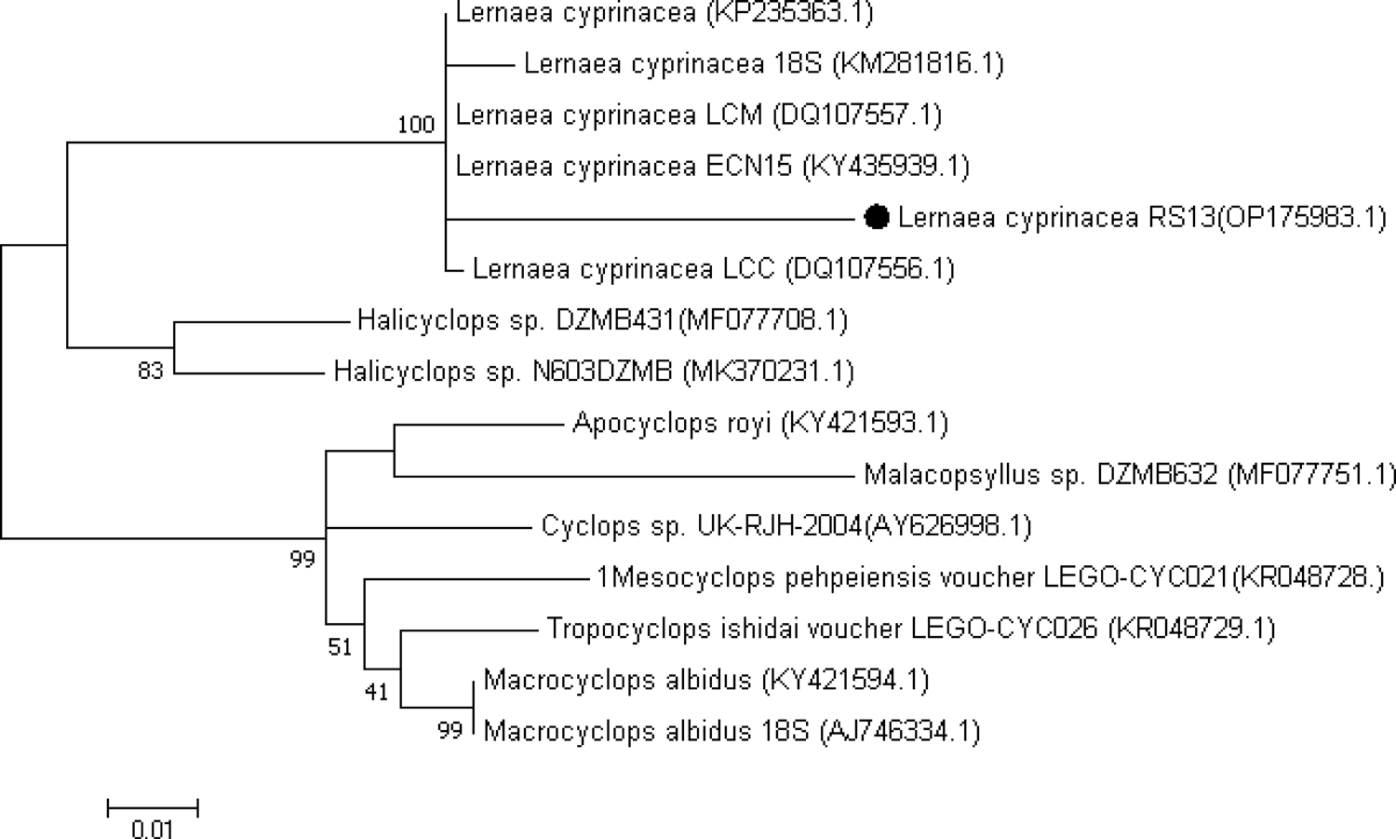
Molecular phylogenetic analysis based on the nucleotide sequences of the partial 5.8 s rDNA gene using the maximum likelihood method based on the general time-reversible model^44^. Evolutionary analyses were performed in MEGA7^43^. The bootstrap consensus tree derived from 1000 replicates represents the evolutionary history of the taxa analyzed. The percentage of trees in which the associated taxa were clustered together is indicated next to the branches. The tree is drawn to scale, with the length of branches measured in the number of substitutions per site.

The results of the BLAST search conducted on the newly obtained partial 18S rDNA sequences (OP175994 and OP175983) confirm the identification of the epibiont ciliate *E. wuhanensis* and the copepod crustacean *L. cyprinacea* in the examined ornamental fish. The sequences were found to be identical, by original description, to the reference sequences KU869709 and KY435939, respectively, with 100% coverage (Figs. 7 and 8; Tables 2 and 3).

**Table 2:**
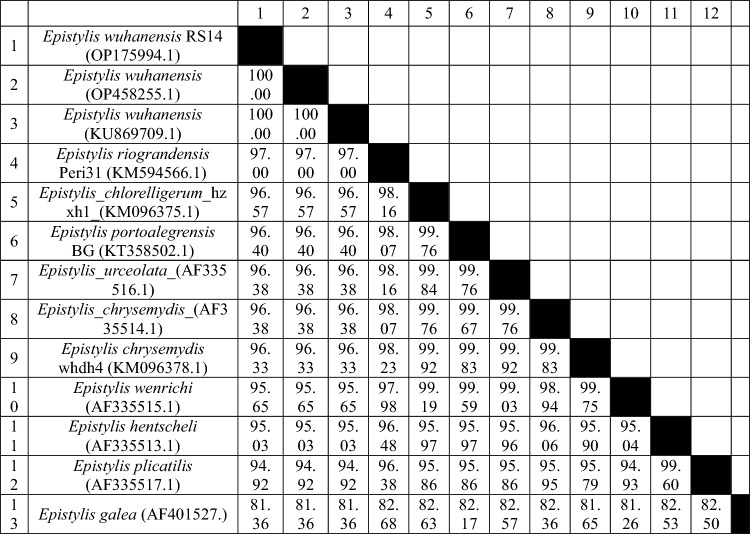
The genetic homology between *Epistylis wuhanensis* and other selected Epistylididae was calculated using the maximum composite likelihood substitution model based on the partial 18 s gene.

**Table 3:**
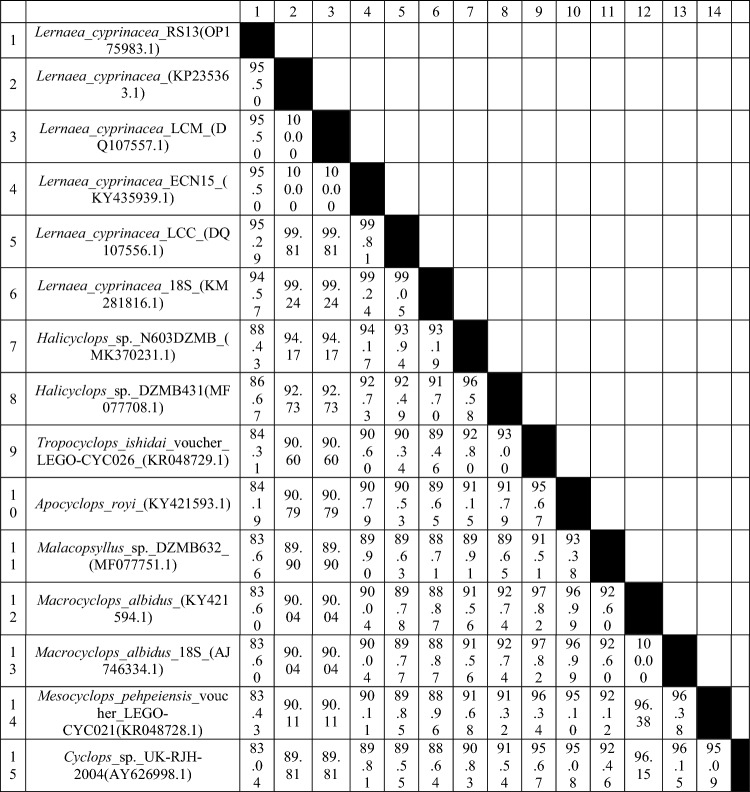
The genetic homology between the *Lernaea cyprinacea* isolate and other selected Lernaeidae was calculated using the maximum composite likelihood substitution model based on the partial 5.8 s rDNA gene.

### Phylogenetic and bioinformatics

The distance analysis of the partial 18S rDNA nucleotide sequences of *E. wuhanensis* obtained in the study revealed 100% identity with the reference sequences OP458255.1 and KU869709.1, both of which are also identified as *E. wuhanensis*. The sequences showed 97% identity with *E. riograndensis* Peri31 (KM594566.1) and 96.57% identity with *E. chlorelligerum* hzxh1 (KM096375.1). This strain was closely related (up to 95%) to *E. portoalegrensis* BG (KT358502.1), *E. urceolata* (AF335516.1), *E. chrysemydis* (AF335514.1), *E. wenrichi* (AF335515.1), and *E. hentscheli* (AF335513.1). According to Wang et al.^27^, this strain demonstrated the close relationship of the clade IV of Epistylididae.

The analysis of *L. cyprinacea* isolates RS13 and OP175994 revealed high identity (up to 95%) with the reference sequences KP235363.1, DQ107557.1, KY435939.1, and DQ107556.1, which are all identified as *L. cyprinacea*. This indicates that the identified *L. cyprinacea* parasites in the examined ornamental fish are closely related to other *L. cyprinacea* strains and are distinct from other *Lernaea* species.

## Discussion

Based on the importance of parasitic infections as one of the most concerning problems affecting ornamental fish^10,45,46^, attempting to gain a better understanding of parasitic infestations affecting ornamental fish may play an important role in the success of the ornamental fish industry^47^. Although the overall prevalence of parasitized fish in the visited ornamental fish farm was not particularly high (20.00%), the presence of detectable parasites at any level is of significance because the majority of parasites recovered from freshwater fish in Iran have the potential to induce significant mortalities among captive and wild stocks, particularly those parasites that do not typically require an intermediate host, such as ciliated protozoans and copepod crustaceans. Therefore, proper diagnosis, treatment, and prevention of parasitic infections are essential for maintaining the health and well-being of ornamental fish in captivity. This includes regular monitoring and screening for parasites, implementing effective biosecurity measures, and providing appropriate water quality and nutrition to reduce stress and susceptibility to parasitic infections. By doing so, ornamental fish farmers can ensure the long-term sustainability and profitability of their business while also promoting the welfare of the fish under their care.

The parasitism of peritrich ciliates has previously been reported as a cause of mortality in North American^48^ and European fish^49^, particularly when combined with opportunistic infections^50,51^. The epistylidid ciliate genus *Epistylis* Ehrenberg, which was described in 1830, contains over 260 nominal species^52^. It is believed that a particular number of species are free-living, but the majority are thought to be epibionts on aquatic metazoans such as aquatic plants, insects, and crustaceans that live in marine and freshwater environments^53^. These epibiont species can occasionally infect fish hosts with diseases such as red-sore disorders^28,54^. On the other hand, crustaceans are important mechanical vectors of various pathogenic agents such as bacteria, viruses, and parasites^55^, and their spread in aquatic environments may have a significant negative impact on commercial fish farming^17,56^. There are only a few reports that show a vectorial relationship between the isopod *Gnathiid* and the haemoprotozoan *Haemogregarina*^57^, as well as the crustacean arthropod *Argulus foliaceus* and *Rhabdovirus carpio*^17,58^, *Caligus rogercresseyi* and ISAV (infectious salmon anemia virus)^59,60^, *Ergasilus chelangulatus* and *Epistylis* spp.^31^, *Acartia bifilosa* and *Epistylis* sp.^61^, *Dolops carvalhoi* and *Epistylis* sp.^17,62^, *L. cyprinacea* and *E. wuhanensis*^27,29^, and *L. cyprinacea* and *Epistylis* sp.^17^. Chronic stress and susceptibility to a wide range of infestations may be increased in fish exposed to crustaceans for an extended period of time due to physical and/or environmental immunosuppressive stressors^63,64^. This, in turn, may pose a significant challenge to the global aquaculture industry by affecting fish health.

Objective histopathological findings in our study confirmed that the invasion of the parasitic crustacean *L. cyprinacea* is capable of causing a wide range of microscopic lesions, including hemorrhage and the aggregation and infiltration of inflammatory cells in the host, which were consistent with the findings of Singh et al.^65^, Hemaprasanth et al.^66^, Pala et al.^17^, and Furtado et al.^67^. Furthermore, parasitic granulomatous inflammation was observed, which agreed with the findings of Furtado et al.^67^. However, alterations reported in the current parasitized fish, such as muscular necrosis and pressure atrophy of skeletal muscles, may be considered novel pathological findings. Because of intraspecific morphological variations, it is difficult to identify sessilids such as *Epistylis* using only clinical and histopathological evaluation without using molecular methods^68,69^. Sequences generated from *E. wuhanensis* specimens and *L. cyprinacea* samples from current study cases are either identical or recovered as sisters to sequences from Japan^29^, implying that they naturally range in or have potentially been transported between Iran and Japan.

In the current study, poor quarantine measures when importing new fish appear to be the main cause of infection caused by *L. cyprinacea* and *E. wuhanensis*. To prevent the spread of parasitic infections in ornamental fish farms, it is essential to implement effective biosecurity measures. This includes extending the quarantine period for newly imported fish and closely monitoring them during this period to detect any potential infections. Fish farmers should also consider dividing their farm into isolation units to prevent the spread of infections between fish populations. Using a specific-pathogen-free (SPF) water source can also help reduce the risk of introducing new parasites to the farm. Additionally, incorporating immunostimulant compounds such as probiotics and prebiotics into fish feed formulations can boost the immune system of the fish and reduce their susceptibility to parasitic infections. Overall, implementing these preventative measures can have significant impacts on disease prevention and control in ornamental fish farms. By reducing the risk of parasitic infections, fish farmers can ensure the health and well-being of their fish populations, as well as the long-term sustainability and profitability of their business.

## Conclusions

The crustacean *L. cyprinacea* serves as a mechanical vector for *E. wuhanensis* infection and spreads the disease in ornamental fish farming operations. For the first time in Iran, we presented diagnostic morphological and molecular findings for sessilids isolated from *L. cyprinacea*. Based on the findings of the current study, such parasitic infections may cause significant economic losses following invasion of the integument area of fish, eventually leading to mass mortalities if treatment is neglected or inadequate. However, further research is needed to determine the precise mechanisms of crustacean attachment and interactions between hosts, crustaceans, and adhered peritrich protozoans. Furthermore, serious consideration should be given to the direct and indirect effects of various environmental factors on the emergence and spread of the current disorder.

## Data Availability

The datasets generated and/or analyzed during the current study are available in the [BankIt] repository [the accession numbers are OP175983 for *Lernaea cyprinacea* isolate RS13 and OP175994 for *Epistylis wuhanensis* isolate RS14].

## References

[1] Abidi R, Khan GE, Chauhan UK (2011). Monogenean infestations among freshwater ornamental fishes: An overview. J. Ecophysiol. Occup. Health..

[2] Faria PMC, Ribeiro K, Almeida CF, Santos FWM, Santos RFB (2016). Aquicultura ornamental: Um mercado promissor. Panorama Aquicult..

[3] Rahmati-Holasoo H, Ahmadivand S, Marandi A, Shokrpoor S, Palić D, Jahangard A (2022). Identification and characterization of lymphocystis disease virus (LCDV) from Indian glassy fish (*Parambassis*
*ranga* Hamilton, 1822) in Iran. Aquacult. Int..

[4] Dey VK (2016). The global trade in ornamental fish. Infofish. Int..

[5] Mousavi HAE (2003). Parasites of ornamental fish in Iran. Bulle. Eur. Assoc. Fish Pathol..

[6] Rahmati-Holasoo H, Marandi A, Mousavi HE, Azizi A (2022). Isolation and identification of *Capillaria* sp. in ornamental green terror (*Andinoacara*
*rivulatus* Günther, 1860) farmed in Iran. Bull. Eur. Assoc. Fish Pathol..

[7] Rahmati-Holasoo H, Marandi A, Ebrahimzadeh MH (2022). The Comprehensive Guide to Goldfish.

[8] Rahmati-Holasoo H, Ghalyanchilangeroudi A, Kafi ZZ, Marandi A, Shokrpoor S, Imantalab B (2023). Detection of lymphocystis disease virus (LCDV) from yellowbar angelfish (*Pomacanthus*
*maculosus* Forsskål, 1775) in Iran: Histopathological and phylogenetic analysis. Aquaculture.

[9] Shokrpoor S, Rahmati Holasoo H, Soroori S, Marandi A, Imantalab B (2022). Basal cell carcinoma in an albino pindani (*Chindongo*
*socolofi*) and a cobalt-zebra (*Maylandia*
*callainos*): Diagnostic imaging, clinical and histopathological study. J. Fish Dis..

[10] Jerônimo GT, Marchiori NDC, Pádua SBD, Dias Neto J, Pilarski F, Ishikawa MM (2012). *Trichodina*
*colisae* (Ciliophora: Trichodinidae): New parasite records for two freshwater fish species farmed in Brazil. Rev. Bras. Parasitol. Vet..

[11] Lieke T, Meinelt T, Hoseinifar SH, Pan B, Straus DL, Steinberg CE (2023). Sustainable aquaculture requires environmental-friendly treatment strategies for fish diseases. Rev. Aquac..

[12] Meshgi B, Eslami A, Yazdani H (2006). Study on the parasitic infections of aquarium fishes around Tehran. J. Fac. Vet. Med..

[13] Mousavi HE, Behtash F, Rostami-Bashman M, Mirzargar SS, Shayan P, Rahmati-Holasoo H (2011). Study of *Argulus* spp. infestation rate in Goldfish, *Carassius*
*auratus* (Linnaeus, 1758) in Iran. Human Vet. Med..

[14] Roberts H (2011). Fundamentals of Ornamental Fish Health.

[15] Moravec F (1994). Parasitic Nematodes of Fresh Water Fishes of Europe.

[16] Barber I, Hoare D, Krause J (2000). Effects of parasites on fish behavior: A review and evolutionary perspective. Rev. Fish Biol. Fish..

[17] Pala G, Farias THV, Alves LDO, Pilarski F, Hoppe EGL (2018). Association of *Epistylis* spp (Ciliophora: Peritrichia) with parasitic crustaceans in farmed piava *Megaleporinus*
*obtusidens* (Characiformes: Anostomidae). Rev. Bras. Parasitol. Vet..

[18] Barber I (2007). Parasites, behaviour and welfare in fish. Appl. Anim. Behav. Sci..

[19] Jithendran KP, Natarajan M, Azad IS (2008). Crustacean parasites and their management in brackishwater finfish culture. Mar. Finfish Aquac. Netw..

[20] Luque JL, Pavanelli G, Vieira F, Takemoto R, Eiras J (2013). Checklist of Crustacea parasitizing fishes from Brazil. Check List.

[21] Tavares-Dias M, Dias-Júnior MBF, Florentino AC, Silva LMA, Cunha ACD (2015). Distribution pattern of crustacean ectoparasites of freshwater fish from Brazil. Rev. Bras. Parasitol. Vet..

[22] Ho J (1998). Cladistics of the Lernaeidae (Cyclopoida), a major family of freshwater fish parasites. J. Mar. Syst..

[23] Lester R, Hayward C, Woo PTK (2006). Phylum arthropoda. Fish Diseases and Disorders: Protozoan and Metazoan Infections.

[24] Goodwin AE (1999). Massive *Lernaea cyprinacea* infestations damaging the gills of channel catfish polycultured with bighead carp. J. Aquat. Anim. Health.

[25] Kir I (2007). The effects of parasites on the growth of the crucian carp (*Carassius*
*carassius* L., 1758) inhabiting the Kovada Lake. Turkiye parazitolojii dergisi.

[26] van As JG, Viljoen SA (1984). taxonomic study of sessile peritrichs (Ciliophora: Peritricha) associated with crustacean fish ectoparasites in South Africa. South Afr. J. Zool..

[27] Wang Z, Zhou T, Guo Q, Gu Z (2017). Description of a new freshwater ciliate Epistylis wuhanensis n. sp. (Ciliophora, Peritrichia) from China, with a focus on phylogenetic relationships within family Epistylididae. J. Eukaryot. Microbiol..

[28] Ksepka SP, Bullard SA (2021). Morphology, phylogenetics and pathology of “red sore disease” (coinfection by Epistylis cf. wuhanensis and Aeromonas hydrophila) on sportfishes from reservoirs in the South-Eastern United States. J. Fish Dis..

[29] Nitta M (2022). First Japanese record of *Epistylis*
*wuhanensis* (Ciliophora: Epistylididae) Attached to *Lernaea*
*cyprinacea* (Copepoda), with a List of *Epistylis* species attached to Metazoans in Japan. Spec. Divers..

[30] Silva RJD, Azevedo RKD, Abdallah VD (2011). First record of an epibiont protozoan *Epistylis* sp. (Ciliophora, Peritrichia) attached to *Amplexibranchius*
*bryconis* Thatcher & Paredes, 1985 (Copepoda, Ergasilidae) from Peixe’s River, state of São Paulo, Brazil. Crustaceana.

[31] Azevedo RK, Brandão H, Abdallah VD, Silva RJD (2014). First record of an epibiont protozoan *Epistylis* sp. (Ciliophora, Peritrichia) attached to *Ergasilus*
*chelangulatus* (Ergasilidae) in Brazil. Braz. J. Biol..

[32] Corrêa LL, Oliveira MSB, Prestes L, Tavares-Dias M (2016). First record in Brazil of *Epistylis* sp. (Ciliophora) adhered to *Argulus* sp. (Argulidae), a parasite of *Hoplias*
*aimara* (Eritrhinidae). Nat. Resour..

[33] Christensen-Dalsgaard KK, Fenchel T (2003). Increased filtration efficiency of attached compared to free-swimming flagellates. Aquat. Microb. Ecol..

[34] Shimeta J, Starczak VR, Ashiru OM, Zimmer CA (2001). Influences of benthic boundary-layer flow on feeding rates of ciliates and flagellates at the sediment-water interface. Limnol. Oceanogr..

[35] Wahl M (1989). Marine epibiosis. I. Fouling and antifouling: Some basic aspects. Mar. Ecol. Prog. Ser..

[36] Regali-Seleghim MH, Godinho MJ (2004). Peritrich epibiont protozoans in the zooplankton of a subtropical shallow aquatic ecosystem (Monjolinho Reservoir, São Carlos, Brazil). J. Plankton Res..

[37] Southgate P (1994). Laboratory diagnosis of fish disease. In Pract..

[38] Wildgoose W (1998). Skin disease in ornamental fish: Identifying common problems. In Pract..

[39] Damaree RS (1967). Ecology and external morphology of *Lernaea*
*cyprinacea*. Am. Midl. Nat..

[40] Utz LR, Simao TL, Safi LS, Eizirik E (2010). Expanded phylogenetic representation of genera Opercularia and Epistylis sheds light on the evolution and higher-level taxonomy of peritrich ciliates (Ciliophora: Peritrichia). J. Eukaryot. Microbiol..

[41] Yi Z, Song W, Clamp JC, Chen Z, Gao S, Zhang Q (2009). Reconsideration of systematic relationships within the order Euplotida (Protista, Ciliophora) using new sequences of the gene coding for small-subunit rRNA and testing the use of combined data sets to construct phylogenies of the Diophrys-complex. Mol. Phylogenet. Evol..

[42] Hall T. BioEdit version 7.0. 0. *Distributed by the author, website: www. mbio. ncsu. edu/BioEdit/bioedit. html*. Accessed 2004.

[43] Kumar S, Stecher G, Tamura K (2016). MEGA7: Molecular evolutionary genetics analysis version 7.0 for bigger datasets. Mol. Biol. Evol..

[44] Nei M, Kumar S (2000). Molecular Evolution and Phylogenetics.

[45] Rahmati-Holasoo H, Marandi A, Mousavi HE, Taheri MA (2022). Parasitic fauna of farmed freshwater ornamental fish in the northwest of Iran. Aquacult. Int..

[46] Rahmati-Holasoo H, Tavakkoli S, Ebrahimzadeh Mousavi H, Marandi A, Taheri MA (2023). Parasitic fauna of farmed freshwater ornamental sutchi catfish (*Pangasiandon*
*hypophthalmus*) and silver dollar (*Metynnis*
*hypsauchen*) in Alborz province, Iran. Vet. Med. Sci..

[47] Hoshino ÉDM, Hoshino MDFG, Tavares-Dias M (2018). Parasites of ornamental fish commercialized in Macapá, Amapá State (Brazil). Rev. Bras. Parasitol. Vet..

[48] Rogers WA (1971). Disease in fish due to the protozoan *Epistylis* (Ciliata: Peritricha). Proc. Ann. Conf.: Southeast. Assoc. Game Fish Commission..

[49] Zrnčić S, Oraić D, Šoštarić B, Ćaleta M, Bulj I, Zanella D (2009). Occurrence of parasites in Cobitidae from Croatian rivers draining into two different watersheds. J. Appl. Ichthyol..

[50] Esch GW, Hazen TC, Dimock RV, Gibbons JW (1976). Thermal effluent and the epizootiology of the ciliate *Epistylis* and the bacterium *Aeromonas* in association with centrarchid fish. Trans. Am. Microsc. Soc..

[51] Miller RW, Chapman WR (1976). *Epistylis* and *Aeromonas*
*hydrophila* infections in fishes from North Carolina reservoirs. Progress. Fish-Culturist..

[52] Lu B, Shen Z, Zhang Q, Hu X, Warren A, Song W (2020). Morphology and molecular analyses of four epibiotic peritrichs on crustacean and polychaete hosts, including descriptions of two new species (Ciliophora, Peritrichia). Eur. J. Protistol..

[53] Sládecek V (1986). Indicator value of the genus *Epistylis* (Ciliata). Arch. Hydrobiol..

[54] Basson L, van As J, Woo PTK (2006). Trichodinidae and other ciliophorans (Phylum Ciliophora). Fish Diseases and Disorders, Volume 1: Protozoan and Metazoan Infections.

[55] Xu DH, Shoemaker CA, Klesius PH (2007). Evaluation of the link between gyrodactylosis and streptococcosis of Nile tilapia, *Oreochromis*
*niloticus* (L.). J. Fish Dis..

[56] Overstreet RM, Jovonovich J, Ma H (2009). Parasitic crustaceans as vectors of viruses, with an emphasis on three penaeid viruses. Integr. Comp. Biol..

[57] Davies AJ, Smit NJ (2001). The life cycle of *Haemogregarina*
*bigemina* (Adeleina: Haemogregarinidae) in South African hosts. Folia Parasitol..

[58] Ahne W (1985). *Argulus*
*foliaceus* L. and *Piscicola*
*geometra* L. as mechanical vectors of spring viraemia of carp virus (SVCV). J. Fish Dis..

[59] Nylund A, Wallace C, Hovland T, Boxshall GA, Defaye D (1993). The possible role of *Lepeophtheirus**salmonis* (Krøyer) in the transmission of infectious salmon anemia. Pathogens of Wild and Farmed Fish: Sea Lice.

[60] Oelckers K, Vike S, Duesund H, Gonzalez J, Wadsworth S, Nylund A (2014). *Caligus*
*rogercresseyi* is as a potential vector for transmission of Infectious Salmon Anaemia (ISA) virus in Chile. Aquaculture.

[61] Visse M (2007). Detrimental effect of peritrich ciliates (*Epistylis* sp.) as epibionts on the survival of the copepod *Acartia*
*bifilosa*. Proc. Estonian. Acad. Sci. Biol. Ecol..

[62] Pagliarini CD, Franceschini L, Ribeiro CDS, Delariva RL, Amorim JPDA, Ramos IP (2019). Dolops carvalhoi as a vector of *Epistylis* sp between cultivated and wild specimens of *Oreochromis*
*niloticus* in Brazil. Rev. Bras. Parasitol. Vet..

[63] Walker PD, Flik G, Bonga SW (2004). The biology of parasites from the genus *Argulus* and a review of the interactions with its host. Symp. Soc. Exp. Biol..

[64] Tort L (2011). Stress and immune modulation in fish. Dev. Comp. Immunol..

[65] Singh R, Raghavendra A, Sridhar N, Raghunath MR, Eknath AE (2011). Comparative susceptibility of carp fingerlings to *Lernaea*
*cyprinacea* infection. Vet. Parasitol..

[66] Hemaprasanth KP, Sridhar N, Raghuanth MR (2017). *Lernaea cyprinacea* infection in a new host *Puntius*
*pulchellus* in intensive culture system and its control by doramectin. J. Parasit. Dis..

[67] Furtado WE, Cardoso L, Figueredo AB, Marchiori NC, Martins ML (2019). Histological and hematological alterations of silver catfish *Rhamdia*
*quelen* highly parasitized by *Lernaea*
*cyprinacea*. Dis. Aquat. Org..

[68] Jiang C, Shi X, Liu G, Jiang Y, Warren A (2016). Morphology and molecular phylogeny of two freshwater peritrich ciliates, *Epistylis*
*chlorelligerum* Shen (1980) and *Epistylis*
*chrysemydis* Bishop and Jahn (1941) (Ciliophora, Peritrichia). J. Eukaryot. Microbiol..

[69] Kühner S, Simão TL, Safi LS, Gazulha FB, Eizirik E, Utz LR (2016). *Epistylis*
*portoalegrensis* n. sp. (Ciliophora, Peritrichia): a new freshwater ciliate species from southern Brazil. J. Eukaryot. Microbiol..

